# Mineral and bone disorder after kidney transplantation: a single-center cohort study

**DOI:** 10.1080/0886022X.2023.2210231

**Published:** 2023-05-15

**Authors:** Li Sun, Zijie Wang, Ming Zheng, Zhou Hang, Jiawen Liu, Xiang Gao, Zeping Gui, Dengyuan Feng, Dongliang Zhang, Qianguang Han, Shuang Fei, Hao Chen, Jun Tao, Zhijian Han, Xiaobing Ju, Min Gu, Ruoyun Tan

**Affiliations:** a Department of Urology, The First Affiliated Hospital of Nanjing Medical University, Nanjing, China; b Department of Urology, The Second Affiliated Hospital of Nanjing Medical University, Nanjing, China

**Keywords:** Kidney transplantation, mineral and bone disorder, osteoporosis, hyperparathyroidism

## Abstract

**Background:**

The assessment and prevention of mineral and bone disorder (MBD) in kidney transplant recipients (KTRs) have not been standardized. This study aimed to evaluate MBD one year after kidney transplantation (KT) and identify the influencing factors of MBD.

**Methods:**

A total of 95 KTRs in our center were enrolled. The changes in bone mineral density (BMD) and bone metabolism biochemical markers, including serum calcium (Ca), phosphorus(P), 25-hydroxyvitamin D(25(OH)vitD), intact parathyroid hormone (iPTH), bone alkaline phosphatase, osteocalcin (OC), type I collagen N-terminal peptide and type I collagen C-terminal peptide (CTx), over one year after KT were assessed. The possible influencing factors of BMD were analyzed. The relationships between bone metabolism biochemical markers were evaluated. The indicators between groups with or without iPTH normalization were also compared.

**Results:**

MBD after KT was manifested as an increased prevalence of hypophosphatemia and bone loss, persistent 25(OH)vitD deficiency, and partially decreased PTH and bone turnover markers (BTMs). Femoral neck BMD was positively correlated with body mass index (BMI) and postoperative 25(OH)vitD, and negatively correlated with postoperative PTH. Lumbar spine BMD was positively correlated with BMI and preoperative TG, and negatively correlated with preoperative OC and CTx. BMD loss was positively associated with glucocorticoid accumulation. Preoperative and postoperative iPTH was negatively correlated with postoperative serum P and 25(OH)vitD, and positively correlated with postoperative Ca and BTMs. The recipients without iPTH normalization, who accounted for 41.0% of all KTRs, presented with higher Ca, lower P, higher BTMs, advanced age, and a higher prevalence of preoperative parathyroid hyperplasia.

**Conclusions:**

MBD persisted after KT, showing a close relationship with hyperparathyroidism, high bone turnover, and glucocorticoid accumulation.

## 1. Introduction

Kidney transplantation (KT), currently the best treatment strategy for patients in the G5 stage of chronic kidney disease (CKD), can reverse many complications of CKD, and improve the patient’s quality of life and survival rate [1]. As new immunosuppressive agents are being used widely, growing attention has been paid to the non-immune aspects in the post-transplant period, which aims to optimize the long-term health of CKD patients.

Chronic kidney disease-mineral and bone disorder (CKD-MBD), a universal problem in CKD patients, starts early in the disease course and progresses as the patient’s kidney function gets worse. At the G5 stage of CKD, CKD-MBD is mainly characterized by hypocalcemia, hyperphosphatemia, deficiency of 25-hydroxyvitamin D (25(OH)vitD) and 1,25-dihydroxyvitamin D(1,25(OH)vitD), hyperparathyroidism, and an increased fibroblast growth factor 23 (FGF23) level, accompanied with higher risks of cardiovascular disease, fractures, and death [2–4].

After KT, mineral and bone disorder (MBD) will be partially improved as transplanted kidney function recovers. However, with the alleviation of uremic symptoms, enhancement of active vitamin D synthesis, and increase of urinary phosphorus excretion resulting from the response of healthy allografts to elevated parathyroid hormone (PTH) and FGF23, there come *de novo* alterations in mineral metabolism [5–8]. As a result, a considerable number of kidney transplant recipients (KTRs) are afflicted with abnormal bone metabolism, osteoporosis, and even fracture, which decrease the survival rate of transplanted kidneys and the recipients [9,10].

Therefore, MBD after KT has become a chief clinical concern and poses management challenges for clinicians. Yet, few studies have systematically examined MBD after KT. The bone turnover markers in relation to mineral metabolism disturbances and bone densitometry have rarely been evaluated [7,8,11,12]. There lacks standardized assessment of MBD after KT and no consensus has been reached on using bone metabolism biochemical markers to assess MBD in KTRs [2,13].

In this study, the KTRs in our center were followed up for the calcium and phosphorus metabolism regulation indicators, bone turnover markers (BTMs), and bone mineral density (BMD) before and one year after KT. We also analyzed the possible influencing factors of BMD, assessed the relationship between BMD changes and levels of bone metabolism biochemical markers, detected the relationship among bone metabolism biochemical markers, and evaluated the role of PTH in order to better understand the evolution of MBD after KT, aiming to guide the comprehensive evaluation, monitoring, and prevention of MBD in KTRs.

## 2. Materials and methods

### 2.1. Patients and study design

A prospective cohort study was performed on 95 patients receiving the first allograft KT in our center from January 2017 to December 2019. This study was approved by the Ethics Committee of the First Affiliated Hospital of Nanjing Medical University (Ethics Number 2016-SR-029). All patients signed the informed consent form before participating in the study.

All KTRs included were >18 years old with stable postoperative graft function (coefficient of variation of serum creatinine(Scr) <10% in the recent three consecutive measurements). Patients with multiple organ transplantation, pregnancy, malignant tumors, other severe diseases (including severe liver disease, hematologic malignancies, and active infections), or other diseases seriously affecting bone metabolism (such as tumor-related bone diseases, endocrine system diseases, autoimmune diseases, bone dysplasia, long-term bed rest, severe malnutrition, new fractures within 1 year, hip arthroplasty history, compression fracture of the lumbar spine) were excluded.

Data on clinical information, laboratory examination, and imaging examination were collected within 3 months before and one year after KT under the same detecting condition.

### 2.2. Clinical data

Data collected from the KTRs included sex, age, height, weight, body mass index (BMI), menstrual status, dialysis method and dialysis time, primary kidney disease, comorbidities (such as preoperative hypertension, diabetes, etc.), smoking history (continuous or cumulative smoking for 6 months or longer), drinking history (daily alcohol intake of 3 units or more, the unit referring to the WHO fracture risk assessment system), history of total parathyroidectomy with forearm autotransplantation (TPTX + AT), use of immunosuppressant, use of calcium, calcitriol and cinacalcet, as well as glucocorticoid accumulation (calculated based on the dose of methylprednisolone).

After KT, the KTRs’ serum calcium levels were tested every month and calcium supplements was provided to those with hypocalcemia, to maintain their serum calcium levels between 2.2–2.65 mmol/L. The 25(OH)vitD and iPTH levels were tested 3–6 months after KT. The patients with low 25(OH)vitD levels and those with hyperparathyroidism and no hypercalcemia were given ‘calcium carbonate and vitamin D3 tablets’ and calcitriol to maintain their serum 25(OH)vitD levels between 52.5–117.5 nmol/L. As for the glucocorticoid protocol, methylprednisolone was intravenously administered at a dose of 500 mg/day on the day of surgery and until two days after KT; the dosage was reduced thereafter to 400 mg, 300 mg, 200 mg, and then 80 mg/day over each subsequent day, followed by oral administration of 24 mg/day methylprednisolone. The initial immunosuppression protocol for each KTR was methylprednisolone, tacrolimus (FK506, 0.05 to 0.1 mg/kg/day, twice daily reaching a target trough level of 10 ng/mL) or cyclosporine (CsA, 3 to 5 mg/kg/day, twice daily reaching a target trough level of 150 to 250  ng/mL), and mycophenolate mofetil (MMF, 0.75 to 1.0 g twice daily). Once the kidney allograft function was stabilized, FK506(target trough level of 6 to 10 ng/mL) or CsA (target trough level of 100 to 200 ng/mL), MMF (0.5 to 1.0 g twice daily), and methylprednisolone (20 mg daily initially and then tapering to 4 mg daily within three months) were administered as maintenance therapy. The KTRs intolerant to FK506 at the target dose were given low-dose sirolimus additionally.

### 2.3. Laboratory measurements

Fasting blood samples of all included KTRs were collected in the morning. Cobas e602 automatic electrochemical luminescence analyzer (Roche, Basel, Switzerland) was employed to measure Scr, total cholesterol (TC), triglyceride (TG), high-density lipoprotein (HDL), low-density lipoprotein (LDL), and albumin (Alb).

The estimated glomerular filtration rate (eGFR) was calculated using the MDRD formula: eGFR = 170 × Scr ^−0.999^×age^−0.176^×BUN ^−0.170^×Alb ^0.318^×(female × 0.762). Chronic kidney disease (CKD) stages were evaluated according to "Guidelines for Quality of Life of Kidney Diseases and Dialysis Patients" (Kidney Disease Outcomes Quality Initiative, K/DOQI): CKD 1 T GFR ≥ 90 mL/(min × 1.73m^2^), CKD 2 T GFR 60–89 mL/(min × 1.73m^2^), CKD 3 T eGFR 30–59 mL/(min × 1.73m^2^), CKD 4 T eGFR 15–29 (ml/(min × 1.73m^2^), and CKD 5 T eGFR < 15 mL/(min × 1.73m^2^).

Bone metabolism biochemical markers involved (1) calcium and phosphorus metabolism regulators, including serum calcium (Ca), phosphorus (P), intact parathyroid hormone (iPTH), 25(OH)vitD, and (2) BTMs, including osteocalcin (OC), bone-specific alkaline phosphatase (BALP), Type I collagen cross-linked N-terminal peptide (NTx), and type I collagen cross-linked C-terminal peptide (CTx). Cobas e602 automatic electrochemical luminescence analyzer (Roche, Basel, Switzerland) was used to measure serum Ca, serum P, 25(OH)vitD, iPTH, BALP, and OC levels. And Cobas e170 automatic electrochemical luminescence analyzer (Roche, Basel, Switzerland) was applied to measure NTx and CTx. Corrected Ca levels were calculated using the following formula: Correction Ca = serum Ca + (40-serum Alb)×0.02. The serum Ca levels in the following text were all corrected Ca levels.

The increase or decrease of the indicators was determined according to the reference range in our laboratory: Ca 2.2–2.65 mmol/L, P 0.81–1.45 mmol/L, 25(OH)vitD 52.5–117.5 nmol/L, iPTH 12–88pg/mL, OC 11–43n/mL; BALP ≤20.1 mmol/L for males, or ≤14.3 mmol/L for premenopausal females, or ≤22.4 mmol/L for postmenopausal females; NTx 16.9–36.4 ng/mL for males, or 15.1 − 30.1 ng/mL for premenopausal females, or 16.3–37.1 ng/mL for postmenopausal females; CTx <0.3 ng/mL for males, or <0.3 ng/mL for premenopausal females, or <0.6 ng/mL for postmenopausal females. The definitions of normal, mildly elevated, moderately elevated, and severely elevated iPTH were iPTH level ≤88pg/mL, 88–300pg/mL, 300–600pg/mL, and >600pg/mL, respectively. iPTH normalization was defined as iPTH level decreasing to the normal reference range(≤88pg/mL).

### 2.4. Imaging studies

GE Logiq E9 Ultrasound Diagnostic Device (GE Healthcare, Milwaukee, WI, USA) was used to evaluate parathyroid hyperplasia.

Dual energy X-ray absorptiometry (DEXA) (Discovery W S/N 85065; Hologic, 35 CrosbyDrive BedFord, USA) was used to determine the BMD of the femoral neck and lumbar spine. Bone density value was expressed in terms of area bone density in g/cm^2^. T value was calculated as the standard deviation obtained by comparing the KTR’s bone density with the average bone density of healthy young people of the same sex. Z value was the standard deviation obtained by comparing the KTR’s bone density with the average bone density of the normal population of the same sex and the same age.

Diagnostic criteria for osteoporosis [14] were as follows: For males ≥50 years and postmenopausal females, those with a T value ≥ −1.0 were regarded as normal, those with −1.0 > T> −2.5 were considered to have osteopenia, and those with T value ≤ −2.5 were considered to have osteoporosis. For males < 50 years old and premenopausal females, those with Z value ≥ −2.0 were regarded as normal, and those with Z value < −2.0 were considered to have osteopenia. Osteoporosis and osteopenia were collectively referred to as bone loss.

For BMD changes, a 2.0% cutoff was applied to define an increase or decrease in BMD scores [15].

### 2.5. Statistical analysis

SPSS 25.0 software was used for data processing. Normally distributed continuous variables were expressed as mean ± standard deviation (±s). Independent sample *t*-test was used for between-group comparison, one-way ANOVA was used for multigroup comparison, and paired sample T test was used to compare indicators before and after KT. Non-normally distributed continuous variables were expressed as median (P25–P75), and Wilcoxon nonparametric test was applied for between-group comparison. Categorical variables were expressed as rate or composition ratio % (frequency), and Chi-square test or Fisher’s exact probability method was used for between-group comparison. Univariate and multivariate linear regression analyses were used to explore the influencing factors of BMD. For the correlation analysis between bone metabolism indicators and other indicators, if the dependent variable was a normally distributed continuous variable, Pearson correlation test was used; if the dependent variable was a non-normally distributed continuous variable or categorical variable, Spearman correlation test was used. Two-sided *p* < 0.05 indicated a statistically significant difference.

## 3. Results

### 3.1. Basic clinical data of KTRs

Basic clinical data of 95 KTRs are shown in Table 1. Among the 95 transplants, 93 were from deceased donors and two were from living donors. FK506 (given to 90 cases, 94.7%) and cyclosporine (CsA, given to 5 cases, 5.3%) were used as calcineurin inhibitors (CNIs). Sirolimus was used in 7.4% (7 cases) of the KTRs. Perioperative immune induction treatments included basiliximab (92.6%, 88 cases) and anti-thymocyte immunoglobulin (ATG, 7.4%, 7 cases). None of the recipients took cinacalcet before and after KT.

**Table 1.** Basic clinical data of enrolled KTRs.

Index	Level	*n* = 95
Age(years)		40.2 ± 10.8
Sex, %(N)	Male	71.6 (68)
	Female	28.4 (27)
Menstrual status, %(N)	Premenopausal	24.2 (23)
	Menopausal	4.2 (4)
Time of RRT(months)		27 (14,48)
Dialysis mode, %(N)		
	Hemodialysis	70.5 (67)
	Peritoneal dialysis	29.5 (28)
Primary kidney disease, %(N)		
	Glomerulonephritis	93.7 (89)
	Anaphylactic purpura nephritis	3.2 (3)
	Polycystic kidney	2.1 (2)
	Interstitial nephritis	1.1 (1)
Pre-operative hypertension, %(N)		82.1 (78)
Pre-operative diabetes, %(N)		9.5 (9)
Smoking, %(N)		7.4 (7)
Alcohol taking, %(N)		5.3 (5)
TPTX + AT , %(N)		4.2 (4)
Preoperative parathyroid hyperplasia/nodule, %(N)		23.1 (22)
Postoperative parathyroid hyperplasia/nodule, %(N)		28.4 (27)
Postoperative calcium use, %(N)		6.3 (6)
Postoperative calcitriol use, %(N)		20.0 (19)
Preoperative BMI (kg/m^2^)		21.2 ± 3.3
Postoperative BMI (kg/m^2^)		21.7 ± 4.0
Postoperative eGFR (mL/min *1.73 m^2^)		74.7 ± 21.3
CKD stage, %(N)		
	CKD 1 T	25.3 (24)
	CKD 2 T	49.5 (47)
	CKD 3 T	25.3 (24)
	CKD 4 T	0 (0)
	CKD 5 T	0 (0)

Abbreviations: KTRs, kidney transplant recipients; RRT, Renal replacement therapy; TPTX + AT, total parathyroidectomy with forearm autotransplantation; BMI, body mass index; eGFR, estimated glomerular filtration rate.

### 3.2. Changes in bone metabolism biochemical markers and BMD in KTRs

One year after KT, serum P and iPTH decreased (*p* < 0.001) (Table 2A). The prevalence of hypocalcemia, hypercalcemia, and hyperparathyroidism decreased(*p* < 0.001); the prevalence of hypophosphatemia increased (*p* < 0.001); the prevalence of patients with low 25(OH)vitD levels was high (69%) both before and after surgery (Tables 2B and 2C).

**Table 2A.** Changes of bone metabolism biochemical markers after KT.

Index	Pre-transplantation	Post-transplantation	*p* Value
Ca(mmol/L)	2.33 ± 0.23	2.37 ± 0.15	0.136
P(mmol/L)	1.85 ± 0.55	0.93 ± 0.18	<0.001
iPTH (pg/mL)	347.21 ± 306.22	100.66 ± 70.33	<0.001
25(OH)vitD(nmol/L)	47.38 ± 29.06	45.70 ± 18.02	0.527
OC(ng/mL)	180.83 ± 100.27	25.74 ± 20.69	<0.001
BALP(U/L)	17.67 ± 13.54	19.09 ± 14.60	0.342
NTx(ng/ml)	319.12 ± 301.32	76.25 ± 79.14	<0.001
CTx(ng/ml)	2.21 ± 1.40	0.76 ± 0.52	<0.001
FN BMD (g/cm^2^)	0.75 ± 0.13	0.69 ± 0.09	0.001
LS BMD (g/cm^2^)	0.97 ± 0.11	0.87 ± 0.28	0.021

Abbreviations: KT, Kidney transplantation; Ca, calcium; P, phosphorus; iPTH, intact parathyroid hormone; 25(OH)vitD, 25-hydroxyvitamin D; OC, osteocalcin; BALP, bone specific alkaline phosphatase; NTx, Type I collagen cross-linked N-terminal peptide; CTx, Type I collagen cross-linked C-terminal peptide; FN BMD: femoral neck bone mineral density; LS BMD: lumbar spine bone mineral density.

Reference values: Ca 2.2–2.65 mmol/L, P 0.81–1.45 mmol/L, 25(OH)vitD 52.5–117.5 nmol/L, iPTH 12–88pg/ mL, OC 11–43n/mL; BALP ≤20.1 mmol/L for male or ≤14.3 mmol/L for premenopausal female or ≤22.4 mmol/L for postmenopausal female , NTx 16.9–36.4 ng/mL for male or 15.1–30.1 ng/mL for premenopausal female or 16.3–37.1 ng/mL for postmenopausal women; CTx <0.3 ng/mL for male or <0.3 ng/mL for premenopausal women or <0.6 ng/mL for postmenopausal women.

Serum OC, NTx and CTx levels were lower than those before KT(*p* < 0.001) (Table 2A). The prevalence of elevated OC (*p* < 0.001), elevated NTx (*p* = 0.005), and elevated CTx (*p* < 0.001) decreased significantly one year after KT (Table 2B).

**Table 2B.** Prevalence of abnormal Ca, P, 25(OH)vitD, and BTMs levels (*n* = 95).

Index	High	Normal	Low
pre-Ca, %(N)	22.10 (21)	69.50 (66)	8.40 (8)
post-Ca, %(N)	12.60 (12)	82.10 (78)	5.30 (5)
pre-P, %(N)	4.30 (4)	21.30 (20)	74.50 (71)
post-P, %(N)	22.10 (21)	76.80 (73)	1.10 (1)
pre-25(OH)vitD, %(N)	69.50 (66)	29.40 (28)	1.10 (1)
post-25(OH)vitD, %(N)	69.50 (66)	30.50 (29)	0.00 (0)
pre-OC, %(N)	5.50 (5)	8.80 (8)	85.70 (82)
post-OC, %(N)	10.60 (10)	74.70 (71)	14.70 (14)
pre-BALP, %(N)	/	74.70 (71)	25.30 (24)
post-BALP, %(N)	/	69.50 (66)	30.50 (29)
pre-NTx, %(N)	6.30 (6)	3.20 (3)	90.50 (86)
post-NTx, %(N)	6.30 (6)	19.10 (18)	73.7 (70)
pre-CTx, %(N)	/	1.10 (1)	98.90 (94)
post-CTx, %(N)	/	22.10 (21)	77.9 (74)

Abbreviations: Ca, calcium; P, phosphorus; 25(OH)vitD, 25-hydroxyvitamin D; BTMs, bone turnover markers; OC, osteocalcin; BALP, bone specific alkaline phosphatase; NTx, Type I collagen cross-linked N-terminal peptide; CTx, Type I collagen cross-linked C-terminal peptide.

**Table 2C.** Prevalence of abnormal iPTH levels (*n* = 95).

Index	Normal	Mildly Increased	Moderately Increased	Severely Increased
pre-iPTH, %(N)	17.00 (16)	38.30 (36)	28.70% (28)	16.00 (15)
post-iPTH, %(N)	51.60 (49)	46.20 (44)	2.20 (2)	0.00 (0)

Abbreviations: iPTH, intact parathyroid hormone.

Femoral neck (FN) BMD (*p =* 0.001) and lumbar spine (LS) BMD decreased (*p =* 0.021) (Table 2A). The prevalence of FN bone loss (*p =* 0.151) and LS bone loss increased (*p =* 0.042) (Table 2D).

**Table 2D.** Prevalence of abnormal BMD levels (*n* = 95).

Index	Normal	Osteopenia	Osteoporosis
pre-FN BMD, %(N)	78.90 (75)	14.70 (14)	6.30 (6)
post-FN BMD, %(N)	67.40 (64)	29.50 (28)	3.20 (3)
pre-LS BMD, %(N)	81.10 (77)	16.80 (16)	2.10 (2)
post-LS BMD, %(N)	64.20 (61)	31.60 (30)	4.20 (4)

Abbreviations: BMD: bone mineral density; FN BMD: femoral neck bone mineral density; LS BMD: lumbar spine bone mineral density.

### 3.3. Factors influencing BMD in KTRs

#### 3.3.1. Comparison of indexes between groups with increased and decreased BMD after KT

**Figure 1.**
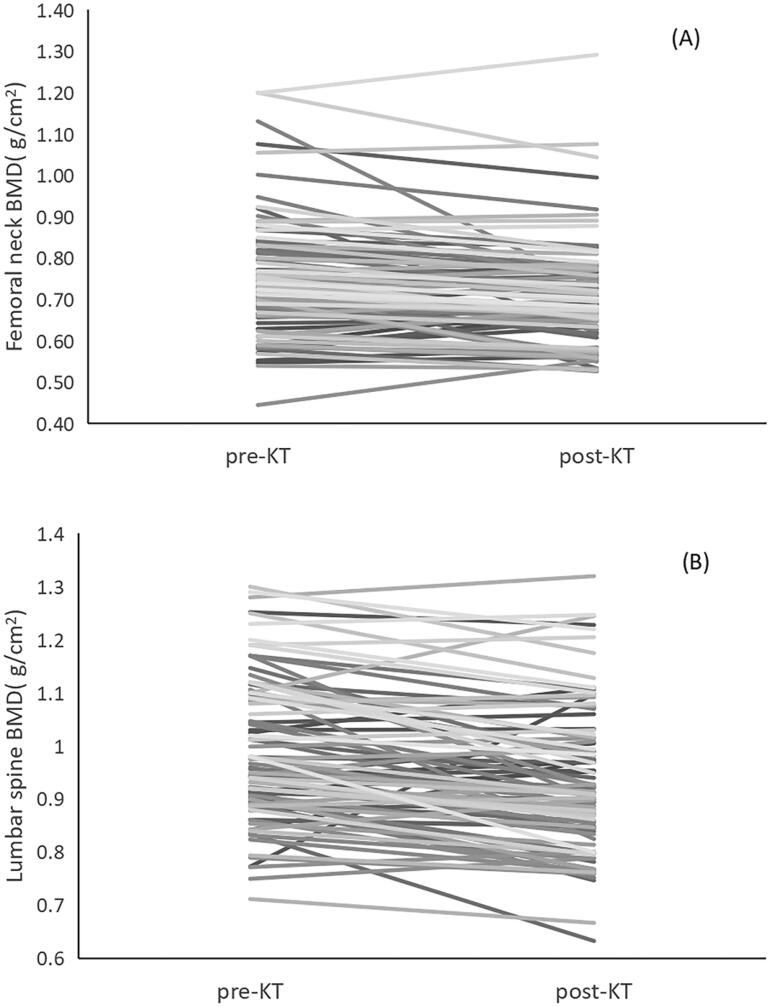
(A) The change trend of individual femoral neck (FS) BMD. (B) The change trend of individual lumbar spine (LS) BMD.

**Figure 2.**
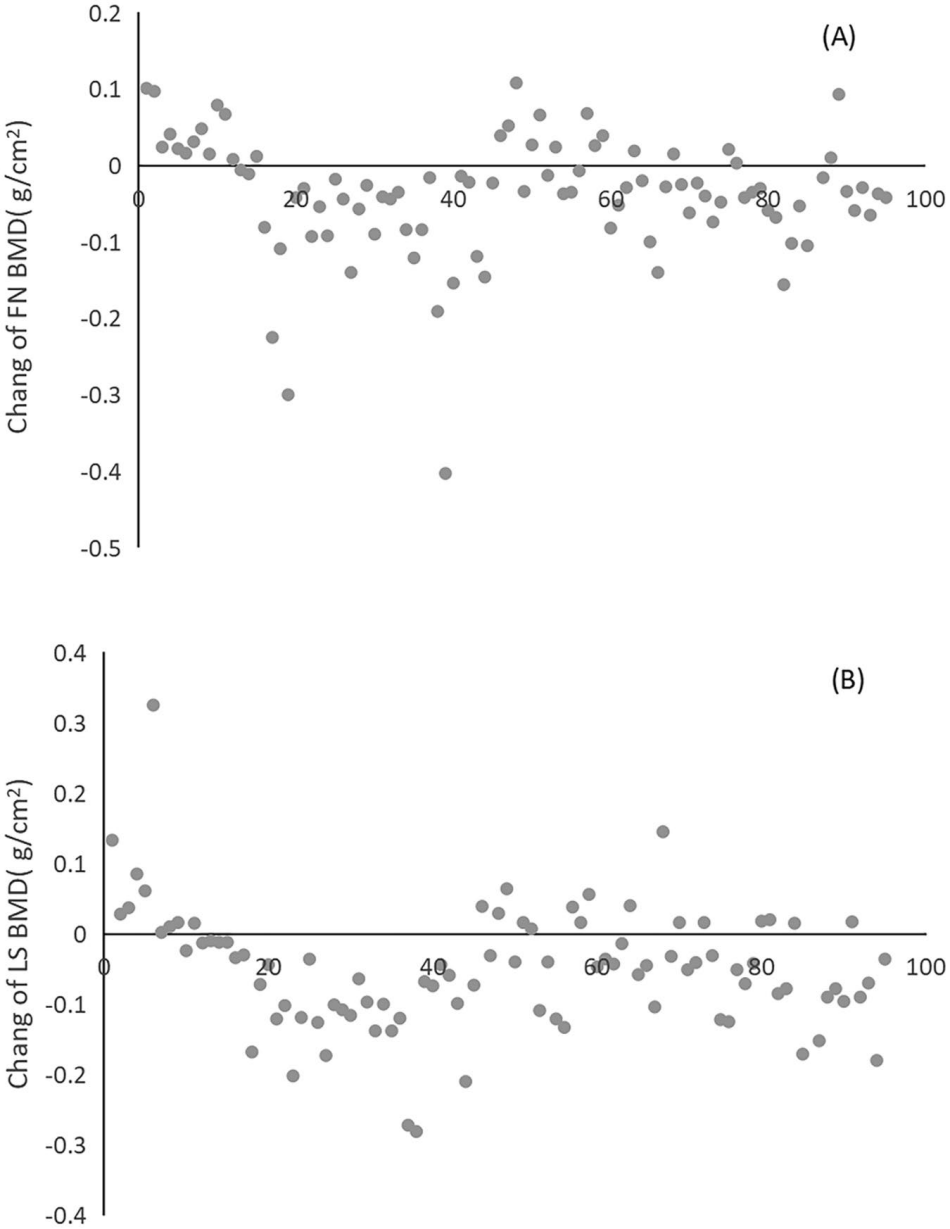
(A) The change value of femoral neck (FS) BMD. (B) The change value of lumbar spine (LS) BMD.

After KT, the FN BMD increased in 23.2% (22 cases) and decreased in 66.3% (63 cases) recipients. LS BMD increased in 13.6% (13 cases) and decreased in 66.3% (63 cases) recipients. The change trend of individual BMD are shown in Figure 1 and the change value of BMD are shown in Figure 2.

Recipients with increased FN BMD had higher postoperative serum Ca (*p =* 0.035) and ΔCa levels (*p* = 0.046). Recipients with increased LS BMD had shorter preoperative dialysis time (*p* = 0.002), higher postoperative TC levels (*p* = 0.032), lower glucocorticoid accumulation (*p* = 0.002) and postoperative CTx levels (*p* = 0.031) (Tables S1 and S2).

#### 3.3.2. Influencing factors of BMD and bone loss in KTRs

Univariate linear regression analysis showed that FN BMD was positively associated with BMI and postoperative 25(OH)vitD, and negatively associated with postoperative iPTH. And these associations were still statistically significant in multivariate analysis. LS BMD was positively associated with BMI and preoperative TG, and negatively associated with preoperative OC and CTx. And the associations with preoperative TG and OC were still statistically significant in multivariate analysis. FN BMD loss and LS BMD loss were both positively associated with glucocorticoid accumulation. No association was found between BTMs changes and BMD loss (Table 3, all demographic and biochemical variables tested, and only significant factors included in the table).

**Table 3.** Results of linear regression analysis of influencing factors of BMD and ΔBMD.

Factors	Univariate analysis			Multivariate analysis		
	Regression coefficient	95%CI	*p* Value	Regression coefficient	95%CI	*p* Value
**Influencing factors of FN BMD**						
BMI (kg/m^2^)	0.012	0.002 ∼ 0.021	0.020	0.010	0.001∼−0.020	0.032
Postoperative 25(OH)vitD(nmol/L)	0.0017	0.0003 ∼ 0.0031	0.002	0.0018	0.0001 ∼ 0.0036	0.039
Postoperative iPTH (pg/mL)	−0.0004	−0.0008∼−0.00005	0.026			
**Influencing factors of LS BMD**						
BMI (kg/m^2^)	0.024	0.004 ∼ 0.043	0.017			
Preoperative TG(mmol/L)	0.039	0.008 ∼ 0.071	0.015	0.035	0.004 ∼ 0.067	0.027
Preoperative OC(ng/mL)	−0.0007	−0.0011∼−0.0002	0.007	−0.001	−0.001∼−0.00016	0.009
Preoperative CTx(ng/mL)	−0.042	−0.077∼−0.007	0.020			
**Influencing factors of FN ΔBMD**						
Glucocorticoid accumulation(g)	−0.0000369	−0.000062∼−0.0000011	0.005			
**Influencing factors of LS ΔBMD**						
Glucocorticoid accumulation(g)	−0.00008248	−0.000162∼−0.000003	0.041			

Abbreviations: FN BMD: femoral neck bone mineral density; LS BMD: lumbar spine bone mineral density; FN ΔBMD, changes of femoral neck bone mineral density; LS ΔBMD, changes of lumbar spine bone bone mineral density; BMI, body mass index; 25(OH)vitD, 25-hydroxyvitamin D; iPTH, intact parathyroid hormone; TG, triglyceride; OC, osteocalcin; CTx, Type I collagen cross-linked C-terminal peptide;.

Annotation: All demographic and biochemical variables have been tested, and only significant factors were included in the table.

### 3.4. Relationship between bone metabolism biochemical markers

#### 3.4.1. Relationship between postoperative bone metabolism biochemical markers and their relationship with preoperative bone metabolism biochemical markers

As for the relationship between postoperative bone metabolism biochemical markers, iPTH was positively correlated with Ca (*r* = 0.471, *p* < 0.001), OC (*r* = 0.359, *p* < 0.001), BALP (*r* = 0.517, *p* < 0.001), NTx (*r* = 0.227, *p* = 0.035), and CTx (*r* = 0.349, *p* = 0.001), and negatively correlated with P (*r*= −0.475, *p* < 0.001) and 25(OH)vitD (*r*= −0.269, *p* = 0.010). BTMs were positively correlated with each other (*p* < 0.05).

Preoperative iPTH was positively correlated with postoperative Ca (*r* = 0.328, *p* = 0.001), iPTH(*r* = 0.293, *p* = 0.005), OC (*r* = 0.315, *p* = 0.002), BALP (*r* = 0.350, *p* = 0.001), NTx (*r* = 0.248, *p* = 0.020), and CTx (*r* = 0.246, *p* = 0.021) and negatively correlated with postoperative P(r= −0.213, *p* = 0.039). That is, postoperative BTMs levels were positively correlated with preoperative and postoperative iPTH (Table S3).

#### 3.4.2. Relationship between Δbone metabolism biochemical markers and their relationship with preoperative bone metabolism biochemical markers

ΔBTMs were positively correlated with each other (*p* < 0.001). ΔPTH was positively correlated with ΔP (*r* = 0.271, *p* = 0.009), ΔOC (*r* = 0.421, *p* < 0.001), ΔBALP(*r* = 0.259, *p =* 0.013), ΔNTx (*r* = 0.285, *p* = 0.008) and ΔCTx (*r* = 0.602, *p* < 0.001), and negatively correlated with ΔCa (r= −0.245, *p =* 0.019).That is, a larger PTH decrease after KT was accompanied by a larger increase in Ca and larger decreases in P and BTMs.

Preoperative iPTH was positively correlated with ΔCa (*r* = 0.287, *p =* 0.005), and negatively correlated with ΔP (*r*= −0.299, *p* = 0.003), ΔPTH (*r*= −0.975, *p* < 0.001), ΔOC (*r*= −0.435, *p* < 0.001), ΔNTx (*r*= −0.334, *p* = 0.001) and ΔCTx (*r* = 0.557, *p* < 0.001) . That is, a higher preoperative iPTH was accompanied by a larger increase in Ca and larger decreases in P, iPTH, and BTMs (Table S4).

### 3.5. Effect of iPTH normalization on bone metabolism after KT and the factors affecting iPTH normalization

#### 3.5.1. Effect of iPTH normalization on bone metabolism

After KT, the iPTH of 41.3% KTRs (38 cases) was restored to the normal level and the iPTH of

41.0% KTRs (39 cases)was not restored to the normal level. Recipients whose iPTH was not restored to the normal level presented higher postoperative Ca (*p =* 0.028), OC (*p =* 0.022), BALP (*p =* 0.001), NTx (*p =* 0.032), CTx (*p =* 0.049), and lower postoperative P (*p* < 0.001). There was no significant difference in BMD between the two groups (Table 4).

**Table 4.** Effect of iPTH normalization on bone metabolism one-year post-KT.

Index	Not normal(39cases)	Normal(38cases)	*p* Value
**Postoperative**			
Ca(mmol/L)	2.41 ± 0.15	2.35 ± 0.13	0.028
P(mmol/L)	0.84 ± 0.14	1.00 ± 0.20	<0.001
iPTH (pg/mL)	147.69 ± 68.62	68.52 ± 51.65	<0.001
25(OH)vitD(nmol/L)	42.08 ± 17.13	48.69 ± 19.53	0.118
OC(ng/mL)	33.29 ± 23.91	21.59 ± 19.86	0.022
BALP(μg/L)	26.60 ± 19.16	14.67 ± 9.50	0.001
NTx(ng/mL)	98.33 ± 76.62	69.67 ± 55.19	0.032
CTx(ng/mL)	0.90 ± 0.54	0.66 ± 0.47	0.049
FN BMD(g/cm^2^)	0.70 ± 0.11	0.72 ± 0.13	0.445
LS BMD(g/cm^2^)	0.93 ± 0.16	0.88 ± 0.30	0.301
**Chang rate(%)**			
ΔCa	3.93(−2.56,8.77)	1.99(−3.35,10.26)	0.603
ΔP	−57.09(−64.08, −46.93)	−60.15(−45.96, −27.92)	0.012
ΔiPTH	−65.67(−75.03, −20.18)	−79.78(−87.02, −67.76)	<0.001
Δ25(OH)vitD	−4.55(−25.60,21.15)	−2.83(−37.26,104.69)	0.589
ΔOC	−88.01(−91.92, −74.11)	−91.20(−93.81, −84.64)	0.034
ΔBALP	30.45(−17.67,82.18)	−6.34(−39.49,35.07)	0.008
ΔNTx	−81.73(−88.18, −67.34)	−80.84(−89.86, −54.72)	0.912
ΔCTx	−65.83(−78.01, −51.85)	−70.89(−87.52, −56.61)	0.141
FN ΔBMD	−2.38(−14,65,3.19)	−4.94(−10.68,3.09)	0.832
LS ΔBMD	−4.61(−12.94, −1.11)	−8.80(−12.68, −0.60)	0.693

iPTH: intact parathyroid hormone; Ca: calcium; P: phosphorus; 25(OH)vitD: 25-hydroxyvitamin D; OC: osteocalcin; BALP: bone specific alkaline phosphatase; NTx: Type I collagen cross-linked N-terminal peptide; CTx: Type I collagen cross-linked C-terminal peptide; FN BMD: femoral neck bone mineral density; LS BMD: lumbar spine bone mineral density; FN ΔBMD: changes of femoral neck bone mineral density; LS ΔBMD: changes of lumbar spine bone bone mineral density.

Reference values: Ca 2.2–2.65 mmol/L, P 0.81–1.45 mmol/L, 25(OH)vitD 52.5–117.5 nmol/L, iPTH 12–88pg/ mL, OC 11–43n/mL; BALP ≤20.1 mmol/L for male or ≤14.3 mmol/L for premenopausal female or ≤22.4 mmol/L for postmenopausal female , NTx 16.9–36.4 ng/mL for male or 15.1–30.1 ng/mL for premenopausal female or 16.3–37.1 ng/mL for postmenopausal women; CTx <0.3 ng/mL for male or <0.3 ng/mL for premenopausal women or <0.6 ng/mL for postmenopausal women.

Annotation: iPTH normalization was defined as iPTH level decreased to normal reference range(≤88pg/mL).

#### 3.5.2. Factors affecting iPTH normalization

Recipients whose iPTH wasn’t restored to the normal level after KT were older (*p =* 0.037) and had a higher incidence of preoperative parathyroid hyperplasia/nodules (*p =* 0.014) (Table S5).

## 4. Discussion

After KT, some recipients suffer abnormal mineral and bone metabolism, which may develop from not only the underlying bone disease before transplantation but also the long-term use of immunosuppressants and the impaired transplanted graft function. Our data indicated that one year after KT, though the prevalence of hypocalcemia, hypercalcemia, and hyperphosphatemia decreased, the prevalence of hypophosphatemia increased to about 22%. The prevalence of 25(OH)vitD deficiency in our study stayed at about 70% both before and after KT, while the prevalence of hyperparathyroidism in our study declined from 80% to 50% one year after KT. Jørgensen et al. [16] reported that at 12 months post-transplant, 56% of patients had persistent hyperparathyroidism, 19% had hypercalcemia, 13% had hypophosphatemia, and 53% had 25(OH)D below 30 ng/ml, respectively, which was consistent with our results. In Ferreira’s study [17], PTH decreased in 89.8% of patients one year after KT. And according to Wolf [11], PTH gradually declined after KT, while approximately 25–80% of recipients had hyperparathyroidism one year after KT, which was in line with our findings.

Our data indicated that the KTRs with abnormal iPTH one year after KT had higher postoperative Ca and lower postoperative P levels, and those with abnormal iPTH after KT showed a higher incidence of preoperative parathyroid hyperplasia/nodules, suggesting the important role of PTH in calcium and phosphorus metabolism after KT. In CKD stage, decreased renal phosphorus excretion resulting from increased bone-derived fibroblast growth factor (FGF-23) and renal insufficiency, together with decreased active vitamin D synthesis, stimulates PTH secretion, which eventually manifests as secondary hyperparathyroidism, vitamin D deficiency, hypocalcemia and hyperphosphatemia [2,18]. Following KT, high PTH and FGF-23 levels increase urinary phosphate excretion and result in hypophosphatemia as the graft function recovers [6]. Enhanced bone reactivity to PTH due to improved uremia symptoms and the effects of high PTH on a functional kidney graft can lead to increased serum calcium levels. If the recipient has early diffuse parathyroid hyperplasia before surgery, the hyperplastic parathyroid glands may regress after KT. Once nodular parathyroid hyperplasia progresses preoperatively, the feedback regulation of PTH may not work well, making the postoperative nodular parathyroid hyperplasia difficult to resolve, and then it may develop into persistent hyperparathyroidism [19,20].

BTMs, which reflect skeletal cell activity, bone metabolism level, and the overall entire skeleton activity, can guide the diagnosis and treatment, and monitor the treatment effects of metabolic bone diseases [21]. However, few studies have been conducted regarding the changes in BTMs in KTRs. Our study showed that one year after KT, the level of BTMs decreased significantly, and the prevalence of elevated OC, NTx and CTx were reduced as well. A follow-up study of KTRs showed that the elevated levels of CTx, NTx, OC, or BALP in CKD G5 stage were significantly reduced one year after KT [11]. In Keronen’s study, no changes in BALP were observed one year after KT [12]. While In Ferreira’s study [17], BALP levels decreased in 68.1% of patients one year after KT. In a prospective cohort study by P. Evenepoel [22], the bone resorption markers decreased significantly in the first year after KT, and the bone formation markers also decreased, but not as sustained and significant as the decrease in resorption markers; meanwhile, the changes in BTMs were correlated with each other and with changes of PTH. Another study analyzing renal osteodystrophy in 141 KTRs one year after transplantation revealed that BTMs were significantly lower in patients with low bone turnover and higher in patients with high turnover [23]. The kidney function may impact the levels of OC, CTx, and NTx as they are cleared through the kidneys [20]. Our correlation analysis between OC, NTx, CTx and Scr before and after KT indicated that the association was not statistically significant (Not shown in the results section). We suppose that it is because all patients in our study were in stage CKD1T-3T that the effect of decreased kidney function on these markers was not obvious. We suggest that bone metabolism markers that are less affected by renal function should be preferred, especially in patients with stage CKD4T-5T.

Our findings indicated that BTMs changes were closely related to hyperparathyroidism after KT. A retrospective study of KTRs also suggested [24] that both PTH and BTMs in the PTH severely elevated group and PTH moderately elevated group showed a downward trend, but the level of BTMs in the PTH severely elevated group was higher than that in the PTH moderately elevated group at each time point after surgery. Therefore, it is considered that KTRs with severe preoperative hyperparathyroidism have higher postoperative BTMs levels. Evenepoel et al. [25] also pointed out that PTH is an independent determinant of BTMs.

In addition, our study showed that FN BMD and LS BMD decreased one year after KT. BMD has been reported to decrease at the first three months after KT and remain essentially unchanged for the following 6 months, while after the first year of KT, it continued to decrease slowly [26,27]. In Evenepoel’s study [22], there was a limited decrease in BMD at various time points in the first year after KT, but with a wide inter-individual variation.

In this study, postoperative FN BMD was negatively correlated with postoperative PTH. LS BMD was negatively correlated with preoperative OC and CTx, and recipients with increased lumbar spine BMD had lower postoperative CTx levels. These results suggested that higher levels of PTH and BTMs before and after KT were important factors of bone loss in KTRs. Hyperparathyroidism mainly affects bone cortical component, the major ingredient of femoral neck. DXA measurement cannot differentiate between the cortical and trabecular component of bone. This may explain the correlation between PTH and FN BMD. No correlation was found between BTMs changes and BMD loss in our study. In studies of non-KTRs, changes of PTH and BTMs were negatively correlated with the changes in BMD [28]. Evenepoel [22] reported that patients with increased BMD had higher preoperative BTMs and lower postoperative BTMs. Iyer et al. [29] suggested that recipients with higher PTH and BTMs levels before KT had more bone loss after KT. Thus, we suggest that dynamic monitoring strategies should be taken when applying BTMs in KTRs. In addition, recipients with increased LS BMD in our study had shorter preoperative dialysis time, indicating that shortening the waiting time of dialysis patients before kidney transplantation is conducive to the recovery of postoperative MBD.

Our results also revealed that recipients with increased LS BMD had lower glucocorticoid accumulation, and BMD loss of FN and LS were both positively correlated with glucocorticoid accumulation. High doses of glucocorticoids can inhibit osteoblast proliferation and differentiation, promote osteoblast and osteocyte apoptosis, and reduce intestinal calcium absorption, thereby causing bone loss [30]. Recent studies regarding the effect of postoperative glucocorticoid withdrawal on BMD have produced mixed results. Farmer et al. [31] showed that after KT, the LS BMD was higher in the glucocorticoid withdrawal group for more than five years compared with those who continued to take prednisone 5.9 mg/day. In Nishioka’s study [30], methylprednisolone was rapidly reduced to 4 mg/day within one month after KT with long-term maintenance, which had minimal impact on bone metabolism and did not increase the risk of acute rejection. In Iyer’s study [29], glucocorticoids were withdrawn three days after KT, and BMD of the distal radius decreased significantly by 12 months, without resulting in decreased BMD of the lumbar spine and hip. Considering that the primary kidney disease of KTRs in China is mostly glomerulonephritis, and glucocorticoid withdrawal after KT may increase the risk of rejection and nephritis recurrence, most researchers in China suggest long-term maintenance of low-dose steroids after KT [32]. In addition, the effects of other immunosuppressants on bone metabolism are still controversial [33–35]. It is suggested that there may be a difference between CNI and mTORi regimens on bones. However, our results did not show the specific effects of calcineurin inhibitors and sirolimus on bone loss, which may be explained by the small number of recipients using sirolimus in this study.

Moreover, our study suggested a positive correlation of BMD with TC and TG levels. A positive correlation between BMD and TG in young females has been previously reported [36]. Jeong et al. [37] found that TC was related to BMD of different bone parts in females. Lipids not only act on the cardiovascular system, but also regulate bone cell metabolism [38]. We suppose that lipid levels may have different dose-dependent effects on bones and the cardiovascular system: moderate levels may benefit bones, while high levels may impair the cardiovascular system. The association of TC and TG with BMD is intriguing, which needs further investigation.

Ours is a prospectively designed follow-up study. The strengths of this study are as follows: first, bone turnover markers in relation to mineral metabolism disturbances and BMD were evaluated extensively; second, the influencing factors of PTH recovery and BMD changes were analyzed comprehensively. There also exist some limitations. First, the sample size was relatively small and it was a monocentric study, which limited the identification of risk factors for bone loss and the generalization of the results to other transplant centers. Second, the follow-up time was short. We did not evaluate the long-term changes in bone metabolism after KT and its relationship with long-term survival. Third, most recipients were of CKD1T-3T stage, and therefore the results could not be representative of bone metabolism in KTRs of CKD4T-5T stage. And we excluded patients with a history of fractures. Since fracture is a most severe complication of CKD-BMD, excluding those patients might cause potential selection bias. Furthermore, bone biopsy, which was considered as the gold standard of CKD-MBD, was not applied in the current study.

In conclusion, one year after KT, MBD can be restored to the normal level in only a fraction of patients. MBD in KTRs is mainly characterized by hypercalcemia, hypophosphatemia, hyperparathyroidism, 25(OH)vitD deficiency, elevated BTMs, and bone loss. Abnormal calcium and phosphorus regulation indexes and the persistence of elevated BTMs are related to hyperparathyroidism before and after KT. Recipients with increased BMD after KT show lower levels of BTMs and PTH, and bone loss is significantly associated with glucocorticoid accumulation. We suggest active treatment of secondary hyperparathyroidism before KT and shortening of the KT waiting time, which is more efficient and economical for both clinicians and KTRs. Long-term dynamic monitoring of MBD is also needed in KTRs, and in-depth exploration of risk factors and related mechanisms of BMD will help improve the long-term management of KTRs.

## Supplementary Material

Supplemental Material

## Data Availability

Reasonable requests for data will be accommodated by contacting the corresponding author.
