# Supplementary material for: Mineral and bone disorder after kidney transplantation: a single-center cohort study
Source: Ren Fail. 2023 May 15;45(1):2210231. doi: 10.1080/0886022X.2023.2210231 (PMC10187110; doi:10.1080/0886022X.2023.2210231)
Supplement: Supplemental Material [file IRNF_A_2210231_SM7281.pdf]

Table S1 Comparison of relevant indexes between groups with increased and decreased BMD

**Index**	**FN BMD increased (22cases)**	**FN BMD decreased (63cases)**	***P*-value**	**LS BMD increased (13 cases)**	**LS BMD decreased (63 cases)**	***P*-value**
Age(years)	42.80±13.03	36.53±7.80	0.225	42.67±10.82	37.43±10.26	0.266
Male , %(N)	77.2(17)	79.4(50)	0.653	84.6(11)	79.4(50)	0.663
Female, %(N)	22.7(5)	20.6(13)	0.653	15.4(2)	20.6(13)	0.663
Premenopausal, %(N)	9.1(2)	20.6(13)		15.4(2)	17.4(11)	
Menopausal, %(N)	13.6(3)	0.0(0)	0.124	0.0(0)	3.2(2)	0.787
Dialysis mode						
Hemodialysis, %(N)	77.2(17)	69.8(44)		84.6(11)	66.7(42)	
Peritoneal dialysis, %(N)	22.7(5)	30.2(19)	0.694	15.4(2)	33.3(21)	0.694
Time of RRT(months)	24.30±17.16	29.20±23.79	0.553	14.33±5.72	31.10±23.45	0.002
Smoking, %(N)	9.1(2)	6.3(4)	1.000	15.4(2)	0.0(0)	1.000
Alcohol taking, %(N)	9.1(2)	3.2(2)	0.561	15.4(2)	3.2(2)	0.288
BMI (kg/m^2)	22.68±2.43	20.67±3.05	0.070	21.57±2.77	21.50±3.34	0.966
DM, %(N)	9.1(2)	6.3(4)	0.544	15.4(2)	9.5(6)	0.571
Immunosuppressor						
FK 506, %(N)	100.0(22)	96.8(61)		100.0(13)	100.0(63)	
CsA, %(N)	0.0(0)	3.2(2)	1.000	0.0(0)	0.0(0)	0.333
Sirolimus, %(N)	0.0(0)	9.5(6)	0.398	0.0(0)	6.3(4)	0.250
eGFR (mL/min *1.73 m^2)	77.47±27.35	72.33±17.04	0.485	77.53±30.33	75.97±17.32	0.861
TC (mmol/L)	5.99±0.84	4.68±0.86	0.320	5.44±0.80	4.60±0.85	0.032
TG (mmol/L)	1.37±0.68	1.42±0.75	0.831	1.41±0.82	1.36±0.60	0.861
TPTX+AT , %(N)	0.0(0)	3.2(2)	0.258	0.0(0)	3.2(2)	0.561
Glucocorticoid accumulation(mg)	4845.00±1038.75	5235.73±958.33	0.281	4136.67±856.45	5170.00±655.58	0.002
Calcium use, %(N)	0.0(0)	0.0(0)	/	0.0(0)	0.0(0)	/
Calcitriol use, %(N)	0.0(0)	3.2(2)	0.258	46.2(6)	31.7(20)	0.329

Abbreviations: FN BMD: femoral neck bone mineral density; LS BMD: lumbar spine bone mineral density; RRT, Renal replacement therapy; BMI, body mass index; DM, diabetes mellitus; FK 506, tacrolimus; CsA, cyclosporine; eGFR, estimated glomerular filtration rate; TC, total cholesterol; TG, triglyceride; TPTX+AT, total parathyroidectomy with forearm autotransplantation.

Information Classification: General

Table S2 Comparison of bone metabolism biochemical markers between groups with increased and decreased BMD

Index	FN BMD increased (22cases)	FN BMD decreased (63cases)	*P*-value	LS BMD increased (13 cases)	LS BMD decreased (63 cases)	*P*-value
**Postoperative**						
Ca (mmol/L)	2.45±0.15	2.36±0.11	0.035	2.40±0.11	2.40±0.12	0.933
P (mmol/L)	0.92±0.24	0.90±0.12	0.797	0.85±0.18	0.92±0.17	0.613
iPTH (pg/mL)	117.20±56.76	86.74±42.30	0.085	88.68±32.33	91.77±46.96	0.842
25(OH)vitD(nmol/L)	48.49±15.30	49.21±17.63	0.908	62.11±29.24	46.09±15.13	0.125
OC(ng/mL)	26.74±18.04	18.95±9.65	0.219	13.27±3.67	23.02±13.04	0.081
BALP(μg/L)	19.53±18.86	14.72±6.74	0.448	11.14±2.62	17.82±12.08	0.192
NTx(ng/mL)	85.00±70.42	49.83±39.56	0.184	33.98±13.36	69.59±52.18	0.144
CTx(ng/mL)	0.84±0.56	0.73±0.45	0.626	0.37±0.20	0.82±0.43	0.031
**Preoperative**						
Ca(mmol/L)	2.22±0.17	2.33±0.24	0.200	2.20±0.22	2.35±0.23	0.180
P(mmol/L)	1.83±0.28	1.97±0.53	0.420	1.98±0.26	1.89±0.60	0.836
iPTH (pg/mL)	456.15±335.50	274.68±317.17	0.131	319.83±92.82	364.34±380.06	0.515
25(OH)vitD(nmol/L)	47.43±28.17	44.63±25.00	0.768	47.61±30.24	46.77±27.24	0.923
OC(ng/mL)	214.70±62.83	198.32±88.43	0.532	147.93±76.54	217.13±86.23	0.078
BALP(μg/L)	16.04±9.33	15.46±14.02	0.904	11.47±1.40	17.90±15.37	0.319
NTx(ng/mL)	519.88±456.01	264.53±160.25	0.114	199.37±111.92	389.05±323.81	0.169
CTx(ng/mL)	2.61±1.38	2.17±1.12	0.318	1.90±0.73	2.54±1.40	0.287
**Chang rate (%)**						
△Ca	11.02（2.41~19.56）	-0.59（-5.39~-0.59）	0.046	6.81（1.00~24.27）	-0.30（-4.43~7.84）	0.146
△P	-48.09（-59.55~-41.85）	-55.40（-63.10~-41.12）	0.396	-59.76（-63.92~-50.59）	-52.34（-62.83~-37.13）	0.327
△iPTH	-69.16（-80.16~-60.05）	-65.57（-76.38~-15.83）	0.413	-74.05（-82.20~-56.14）	-68.91（-75.02~-48.02）	0.508
△25(OH)vitD	3.63（-24.88~77.32）	12.70（-17.22~72.66）	0.701	18.88（-4.64~99.49）	1.56（-31.45~57.77）	0.287
△OC	-89.98（-93.31~-81.88）	-90.22（-93.72~-84.65）	0.623	-90.88（-93.19~-86.17）	-91.14（-93.31~-83.09）	0.915
△BALP	17.13（-15.31~47.37）	8.86（-24.23~33.60）	0.612	-13.38（-22.21~33.78）	12.84（-15.56~49.11）	0.371
△NTx	-81.73（-91.66~-74.99）	-85.12（-89.86~-75.67）	1.000	-81.72（-87.67~-77.93）	-82.67（-91.16~-62.85）	0.851
△CTx	-65.83（-80.45~-56.61）	-70.89（-78.92~-55.61）	0.840	-80.26（-89.09~-62.72）	-65.83（-78.92~-49.60）	0.213

Abbreviations: FN BMD: femoral neck bone mineral density; LS BMD: lumbar spine bone mineral density; Ca, calcium; P, phosphorus; iPTH, intact parathyroid hormone; 25(OH)vitD, 25-hydroxyvitamin D; OC, osteocalcin; BALP, bone specific alkaline phosphatase; NTx, Type I collagen cross-linked N-terminal peptide; CTx, Type I collagen cross-linked C-terminal peptide.

Information Classification: General

Table S3 Relationship between postoperative bone metabolism biochemical markers and preoperative/postoperative bone metabolism biochemical markers

**Postoperative**	**Ca**	**P**	**iPTH**	**25(OH)vitD**	**OC**	**BALP**	**NTx**	**CTx**
**Postoperative**								
Ca	/	/	/		/	/	/	/
P	-0.345**	/	/			/		
iPTH	0.471*	-0.475*	/	/	/	/	/	/
25(OH)vitD			-0.269**	/				
OC	0.402*		0.359*		/	/	/	/
BALP	0.443*	-0.276**	0.517*		0.817*	/	/	/
NTx	0.351**		0.227***		0.915*	0.713*	/	/
CTx	0.408*		0.349**		0.808*	0.731*	0.717*	/
**Preoperative**								
Ca								0.241***
P			0.229***					
iPTH	0.328**	-0.213***	0.293**		0.315**	0.350**	0.248***	0.246***
25(OH)vitD		-0.232***		0.489*				
OC	0.322**		0.303**		0.356*	0.412*	0.284**	0.374*
BALP	0.210***	-0.215***	0.211***		0.441*	0.499*	0.332**	0.263***
NTx	0.346**	-0.272***	0.337**		0.554*	0.520*	0.508*	0.470*
CTx					0.522*	0.438*	0.421*	0.426*

Abbreviations: Ca, calcium; P, phosphorus; iPTH, intact parathyroid hormone; 25(OH)vitD, 25-hydroxyvitamin D; OC, osteocalcin; BALP, bone specific alkaline phosphatase; NTx, Type I collagen cross-linked N-terminal peptide; CTx, Type I collagen cross-linked C-terminal peptide.

*P<0.001，**P<0.01，***P<0.05.

Information Classification: General

Table S4 Relationship between △bone metabolism biochemical markers and their relationship with preoperative bone metabolism biochemical markers

Chang rate	△Ca	△P	△iPTH	△25(OH)vitD	△OC	△BALP	△NTx	△CTx
**Chang rate**								
△Ca	/	/	/		/		/	/
△P	-0.354*	/	/					
△iPTH	-0.245***	0.271**	/		/	/	/	/
△25(OH)vitD				/				
△OC	-0.301**		0.421*		/		/	/
△BALP			0.259***			/		/
△NTx	-0.223***		0.285**		0.491*		/	/
△CTx	-0.300**		0.602*		0.534*	0.342**	0.606*	/
**Preoperative**								
Ca	-0.820*					0.247		0.232***
P	0.216***	-0.950*	-0.305**		-0.209***			-0.217***
iPTH	0.287**	-0.299**	-0.975*		-0.435*		-0.334**	-0.557*
25(OH)vitD				-0.790*				
OC	0.316**	-0.212***	-0.492*		-0.979*		-0.506*	-0.498*
BALP	0.331***		-0.655*		-0.455*	-0.443*	-0.374*	-0.559*
NTx	0.286**		-0.437*		-0.455*		-0.968*	-0.498*
CTx	0.357**		-0.757*		-0.536*		-0.576*	-0.928*

Abbreviations: Ca, calcium; P, phosphorus; iPTH, intact parathyroid hormone; 25(OH)vitD, 25-hydroxyvitamin D; OC, osteocalcin; BALP, bone specific alkaline phosphatase; NTx, Type I collagen cross-linked N-terminal peptide; CTx, Type I collagen cross-linked C-terminal peptide.

$^{*}P<0.001$，$^{**}P<0.01$，$^{***}P<0.05$.

Information Classification: General

Table S5 Factors affecting iPTH normalization one year post-KT

**Index**	**Not normal(39cases)**	**Normal(38cases)**	***P*-value**
Age(years)	43.1±1.1	37.8±11.1	0.037
Male , %(N)	79.5(31)	68.4(26)	0.268
Menopausal, %(N)	2.5(1)	7.9(3)	0.320
Hemodialysis, %(N)	84.6(33)	65.8(25)	0.055
Time of RRT(months)	29.9±20.7	36.9±27.3	0.220
Smoking, %(N)	7.7(3)	2.6(1)	0.615
Alcohol taking, %(N)	7.7(3)	5.3(2)	1.000
BMI(kg/m^2)	22.18±2.78	21.98±5.06	0.825
Immune induction treatments			
Balliximab, %(N)	89.7(35)	97.4(37)	0.450
ATG, %(N)	10.3(4)	2.6(1)	
Immunosuppressor			
FK 506, %(N)	89.7(35)	100.0(38)	0.115
CsA, %(N)	10.3(4)	0.0(0)	
Sirolimus, %(N)	7.7(3)	2.6(1)	0.615
eGFR (mL/min *1.73 m^2)	77.01±23.13	74.69±21.81	0.652
TPTX+AT , %(N)	7.6(3)	7.8(3)	0.615
Preoperative parathyroid hyperplasia/nodules, %(N)	51.2(20)	18.4(7)	0.014
Postoperative parathyroid hyperplasia/nodules, %(N)	38.5(15)	21.1(8)	0.108
Glucocorticoid accumulation(mg)	5092.41±1040.41	5256.53±754.81	0.432
Calcium use, %(N)	0.0(0)	0.0(0)	/
Calcitriol use, %(N)	5.1(2)	13.2(5)	0.263
Preoperative bone metabolism biochemical markers			
Ca(mmol/L)	2.33±0.19	2.29±0.28	0.438
P(mmol/L)	1.96±0.54	1.86±0.58	0.439
iPTH (pg/mL)	426.82±358.27	369.73±231.95	0.410
25(OH)vitD(nmol/L)	47.66±30.68	48.11±28.21	0.948
OC(ng/mL)	206.79±98.13	191.21±82.27	0.464
BALP(μg/L)	20.62±16.92	17.70±11.74	0.394
NTx(ng/mL)	448.14±398.34	314.94±265.55	0.113
CTx(ng/mL)	2.54±1.64	2.43±1.27	0.762
FN BMD(g/cm^2)	0.73±0.14	0.75±0.14	0.602
LS BMD(g/cm^2)	0.98±0.12	0.97±0.11	0.848

Abbreviations: iPTH, intact parathyroid hormone; RRT, Renal replacement therapy; BMI, body mass index; ATG, anti-thymocyte immunoglobulin; FK 506, tacrolimus; CsA, cyclosporine; eGFR, estimated glomerular filtration rate; TPTX+AT, total parathyroidectomy with forearm autotransplantation; Ca, calcium; P, phosphorus; iPTH, intact

Information Classification: General

parathyroid hormone; 25(OH)vitD, 25-hydroxyvitamin D; OC, osteocalcin; BALP, bone specific alkaline phosphatase; NTx, Type I collagen cross-linked N-terminal peptide; CTx, Type I collagen cross-linked C-terminal peptide; FN BMD: femoral neck bone mineral density; LS BMD: lumbar spine bone mineral density.

Annotation: iPTH normalization was defined as iPTH level decreased to normal reference range(≤88pg/mL).

Information Classification: General
